# miR‐451a inhibits cancer growth, epithelial‐mesenchymal transition and induces apoptosis in papillary thyroid cancer by targeting PSMB8

**DOI:** 10.1111/jcmm.14673

**Published:** 2019-09-27

**Authors:** Xinlong Fan, Yuejiao Zhao

**Affiliations:** ^1^ Second Ward of Head & Neck Surgery Liaoning Cancer Hospital & Institute (Cancer Hospital of China Medical University) Shenyang China

**Keywords:** miR‐451a, miRNAs, papillary thyroid cancer, PSMB8

## Abstract

Despite the increasing incidence of papillary thyroid cancer in the past decade, the molecular mechanism underlying its progression remains unknown. Several studies have reported down‐regulation of miR‐451a or circular miR‐451a in papillary thyroid cancer cell lines or patients. However, the underlying molecular mechanism remains unknown. In this study, we found that overexpression of miR‐451a could inhibit proliferation, epithelial‐mesenchymal transition and induce apoptosis in papillary thyroid cancer cells. Proteasome subunit beta type‐8 was predicted to be a direct target of miR‐451a and was validated with a luciferase reporter assay. Further functional assays showed that miR‐451a could inhibit thyroid cancer progression by targeting proteasome subunit beta type‐8.

## 1 INTRODUCTION

The incidence of thyroid cancer increased significantly in the past decade.^1^, ^2^, ^3^ Papillary thyroid cancer (PTC) is the most common histological type of thyroid cancer. PTC patients are usually associated with a rather good prognosis.^4^ Patients with PTC respond positively to the surgical resection and thyroid‐stimulating hormone suppression with a 5‐year survival rate more than 90%.^5^, ^6^ However, the prognosis and clinical surgical treatment do differ significantly between benign thyroid nodule patients and thyroid cancer patients. Usually, patients with benign thyroid nodule only need a limited resection of the thyroid nodule itself to eliminate the cancerous potential. Thyroid cancer patients, on the other hand, need a extensive resection of the cancer tissues, surrounding thyroid tissues and lymph nodes. Most of the thyroid cancer patients underwent surgical resection need to take levothyroxin sodium tablets for the rest of their life due to the loss of the normal endocrine function of their thyroid. Therefore, thyroid cancer patients would benefit greatly from further identification of key molecules which regulate the carcinogenesis of thyroid cancer. These key molecules could be novel therapy targets or early detection biomarkers for thyroid cancer patients and thus allow these patients to obtain a better prognosis with a less traumatic surgery.

microRNAs (miRNAs) belong to small noncoding RNAs which regulate the expression of their target genes at the post‐transcription level.^7^ miRNAs bind to the seed sequences of the 3’‐untranslated region of their target messenger RNAs (mRNAs) and mediate the degradation of corresponding mRNAs or inhibit their translation.^8^ Multiple studies have reported the correlation between aberrant expression of miRNAs and cancer progression.^9^, ^10^, ^11^, ^12^ Furthermore, free miRNAs could be detected in human body fluids like serum. The expression profile of cancer patients’ serum miRNAs differs significantly from healthy people's expression profile.^13^, ^14^, ^15^ This makes serum miRNAs promising candidates of novel biomarkers for cancer detection.^16^ Several studies have reported the close correlation between miR‐451a and PTC progression. The expression level of miR‐451a was reported to be lower in PTC cell lines and cancer tissues.^17^, ^18^ miR‐451a was also reported to be lower in PTC tissues from patients with lymph node metastasis than patients without lymph node metastasis, and circulating miR‐451a was reported to serve as a novel biomarker for PTC.^18^, ^19^ Finally, miR‐451a was reported to inhibit the proliferation of PTC cell lines in vitro.^17^ However, the underlying molecular mechanism of the cancer suppressor functions of miR‐451a still remains unknown.

In this study, we found that miR‐451a could inhibit the proliferation of PTC cell lines both in vitro and in vivo. Furthermore, we found that overexpression of miR‐451a induced apoptosis and inhibited epithelial‐mesenchymal transition in PTC cell lines in vitro. Proteasome subunit beta type‐8 (PSMB8) was predicted to be a direct target of miR‐451a and was validated with a luciferase reporter assay. Finally, cellular functional experiments indicated that miR‐451a performs these aforementioned biological functions by targeting PSMB8.

## 2 MATERIALS AND METHODS

### 2.1 Ethics approval and consent to participate

Animal study in this study was approved by the ethics committee of the Affiliated Cancer Hospital of China Medical University (Liaoning Cancer Hospital and Institute). All animal handling procedures in this study were performed in accordance with the standard requirements of the local ethics committee.

### 2.2 Cell lines and cell culture

Human papillary thyroid cancer cell lines B‐CPAP and KTC‐1 were purchased from SIBCB (Shanghai Institute of Biochemistry and Cell Biology, Chinese Academy of Sciences) and used in this study. Cells were cultured in RPMI‐1640 (Gibco‐BRL), supplemented with 10% foetal bovine serum (Bioserum), 100 U/mL penicillin G and 100 μg/mL streptomycin.

### 2.3 Reverse transcription‐quantitative polymerase chain reaction

The total RNA of cell lines was isolated using TRIzol reagent (Invitrogen) according to the manufacturer's protocol. Concentration of isolated total RNA was determined using a NanoDrop 2000 spectrophotometer (Thermo Fisher Scientific). For miR‐451a detection, total RNA was converted into cDNA by using the miRNA 1st‐strand cDNA synthesis kit (Vazyme) and further subjected to quantitative PCR using the miRNA SYBR qPCR Master Mix kit (Vazyme). For proteasome subunit beta type‐8 detection, total RNA was converted into cDNA by the RT SuperMix for qPCR kit (Vazyme) and further subjected to qPCR using the SYBR qPCR Master Mix KIT (Vazyme). U6 snRNA and GAPDH were used as internal controls for miR‐451a and proteasome subunit beta type‐8, respectively. Sequences of primers that were used in this study are provided as below. Hsa‐miR‐451a forward: 5′‐ACACTCCAGCTGGGAAACCGTTACCATTAC‐3′, reverse: 5′‐CTCAACTGGTGTCGTGGAGTCGGCAATTCAGTTGAGCTTACAG‐3′, U6 forward: 5′‐CGCTTCGGCAGCACATATAC‐3′, reverse: 5’‐TTCACGAATTTGCGTGTCATC‐3′, PSMB8 forward: 5′‐GCTGCCTTCAACATAACATCA‐3′, reverse: 5′‐CTGCCACCACCACCATTA‐3′, GAPDH forward: 5′‐ACAACTTTGGTATCGTGGAAGG‐3′, reverse: 5′‐GCCATCACGCCACAGTTTC‐3′.

### 2.4 Western blotting assay

Isolated cells were re‐suspended on ice using RIPA buffer supplemented with protease inhibitor cocktail for 30 minutes. The concentration of the protein was measured by Bicinchoninic Acid Protein Assay Kit (Beyotime) according to manufacturer's protocol. Samples of equivalent total protein (40 μg) were loaded in a polyacrylamide slab gel and transferred to a PVDF membrane. Membranes were incubated with primary antibody against proteasome subunit beta type‐8, E‐cadherin, N‐cadherin, ZO‐1, vimentin, β‐Actin (1:10 000, 1:4000, 1:4000, 1:1000, 1:12 000 and 1:8000, ProteinTech) overnight at 4°C. Blots were then incubated with corresponding secondary antibody (diluted 1:2000, ProteinTech). Protein bands were visualized using enhanced BeyoECL Plus kit (Beyotime).

### 2.5 Transfection experiments

Synthetic miR‐451a mimics, miRNA mimic negative control, miR‐451a inhibitor, negative control RNA inhibitor, proteasome subunit beta type‐8 siRNA, negative control siRNA and overexpression plasmid were purchased from Genepharma Biotechnology (Genepharma). Transfection experiments were performed using Lipofectamine 3000 Reagent (Thermo Fisher Scientific) in accordance with the manufacturer's protocols. In brief, cells were cultured in 6‐well plate until they reached 60%‐70% confluence. About 4 μg of transfection reagents (miR‐451a mimics, miRNA mimic negative control, miR‐451a inhibitor, negative control RNA inhibitor, proteasome subunit beta type‐8 siRNA, negative control siRNA) were added into one well. Cells were collected after 24 hours of co‐culture.

### 2.6 Cell proliferation assay

Cell proliferation was analysed using the Cell Counting Kit‐8 (CCK‐8), the colony formation assay and the Cell‐Light 5‐ethynyl‐2'‐deoxyuridine (EdU) DNA cell proliferation assay. The CCK‐8 (Dojindo) assay was performed in accordance with the manufacturer's protocols. For the colony formation assay, cells were cultured in a 6‐well plate with corresponding experimental treatment (1 × 10^4^ cells per well). Colonies were visualized and analysed after 7 days by staining with crystal violet (Beyotime). For the EdU DNA cell proliferation assay, EdU was added to the culture medium at a ratio of 1:1000 (EdU: culture medium) for 6 hours at 37˚C. Cells with corresponding experimental treatment were fixed in 4% paraformaldehyde for 30 minutes at room temperature and treated with 0.5% Triton‐X for 30 minutes at 37˚C before being incubated with an Apollo reaction cocktail in the dark at room temperature for 1 hours. Nuclei were stained with Hoechst 33342, and cells were observed under a fluorescence microscope. Exposure times were 28 seconds for visualization of Apollo and 0.175 seconds for nuclei.

### 2.7 Flow cytometric analysis for apoptosis assay

For apoptosis analysis, cells were collected after experimental treatment and washed with PBS. Collected cells were stained with the FITC/Annexin V Apoptosis Detection Kit I (BD Biosciences) and further analysed by flow cytometric analysis.

### 2.8 Immunofluorescence assay

Cells were cultured on cover slides with experimental treatment and fixed with 4% paraformaldehyde at room temperature for 20 minutes. Fixed cells were incubated in blocking buffer (5% BSA suspended in PBS) for half an hour before they were incubated with E‐cadherin and N‐cadherin primary antibodies (ProteinTech, 1:100) overnight at 4°C. On the second day, cells were incubated with fluorescein‐conjugated anti‐mouse IgG (ProteinTech, 1:100). The nucleus was stained with DAPI for 3 minutes before the stained cells were observed with a confocal microscope (Nikon).

### 2.9 Luciferase reporter assay

Putative targets of miR‐451a were predicted by TargetScan (http://targetscan.org/), and proteasome subunit beta type‐8 was predicted as a potential target of miR‐451a. The 3′‐UTR of proteasome subunit beta type‐8 containing the wild‐type and mutant miR‐451a binding site was chemically constructed by Genechem biotechnology (Genechem). A total of 3 × 10^4^ B‐CPAP or KTC‐1 cells were seeded in triplicate in 24‐well plates and cotransfected with corresponding plasmids and miR‐451a mimics. Luciferase activity was detected using a dual‐Luciferase reporter assay system (Promega), in accordance with the manufacturer's protocols.

### 2.10 Bioinformatics analysis

The expression profile of PSMB8 in thyroid cancer with the data from The Cancer Genome Atlas was analysed with UALCAN (http://ualcan.path.uab.edu/).^20^ The expression level of PSMB8 in different subgroups based on sample types and histological subtypes was analysed and visualized as bar charts.

### 2.11 Xenograft tumour assay

Subcutaneous xenograft tumour models were generated with 4‐week‐old BALB/c nude mice (Huafukang). A total of 5 × 10^6^ B‐CPAP cells in 150 μL PBS transfected with miR‐451a mimics or miR‐NC were subcutaneously injected into the right axilla area. The size of implanted tumours was measured twice a week with a vernier caliper. Mice were killed at 3 weeks after the initial implantation, and the implanted xenograft tumour tissues were excised. Tumour's volume was measured as: tumour volume = (L × W2)/2, L for tumour long axis, W for tumour short axis.

### 2.12 Statistical analysis

Student's *t* test was performed with SPSS Statistics version 19. *P*‐values of <.05 were considered statistically significant.

## 3 RESULTS

### 3.1 miR‐451a inhibits proliferation and induces apoptosis of papillary thyroid cancer cells

**Figure 1:**
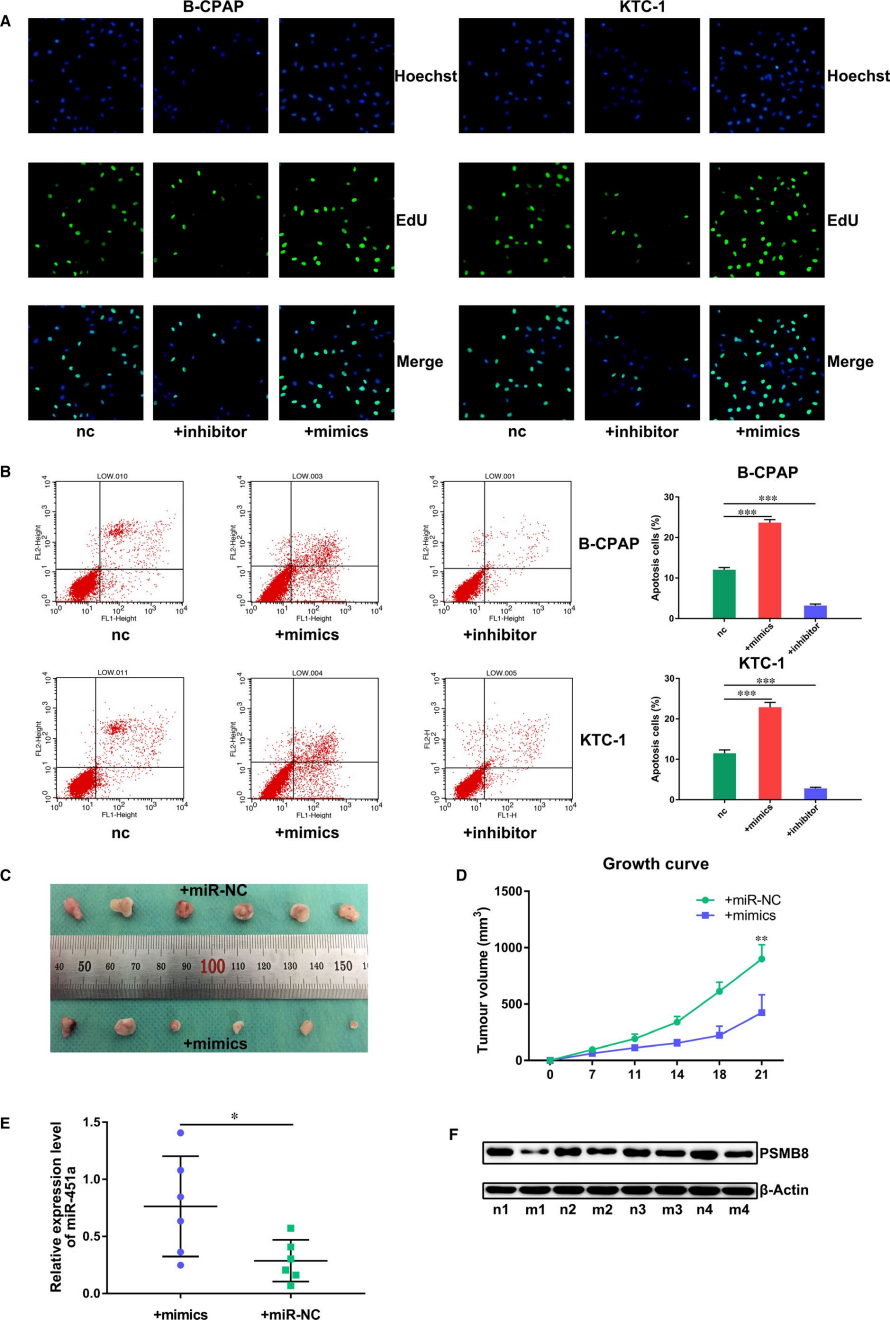
miR‐451a inhibits proliferation and induces apoptosis of papillary thyroid cancer cells. A, EdU DNA cell proliferation assay of B‐CPAP or KTC‐1 cells transfected with miR‐451a mimics or inhibitor. B, Apoptosis assay of B‐CPAP or KTC‐1 cells transfected with miR‐451a mimics or inhibitor. C, Surgically excised xenograft tumour tissues at 3 wk after initial implantation. D, Growth curve of subcutaneously implanted tumours. E, miR‐451a expression in surgically excised tumour tissue by RT‐qPCR. F. PSMB8 expression in surgically excised tumour tissue by Western blotting assay; nc for negative control, n for nude mice treated with miR‐NC, m for nude mice treated with mimics; *P* < .05 marked *, *P* < .01 marked ** and *P* < .001 marked ***

We increased or decreased the expression of miR‐451a in B‐CPAP and KTC‐1 cells by transfection of miR‐451a mimics or inhibitors (Figure S1A). In vitro proliferation assays showed that overexpression of miR‐451a suppressed the proliferation capabilities of B‐CPAP and KTC‐1 cells (Figure 1A, Figure S1B). In vitro apoptosis assay showed that overexpression of miR‐451a induced apoptosis in these two cell lines (Figure 1B). We established subcutaneous tumour models with nude mice to further validate the results of these in vitro experiments in an in vivo setting. The results showed that overexpression of miR‐451a suppressed the growth of transplanted xenograft tumours (Figure 1C‐F).

### 3.2 miR‐451a inhibits epithelial‐mesenchymal transition of papillary thyroid cancer cells

**Figure 2:**
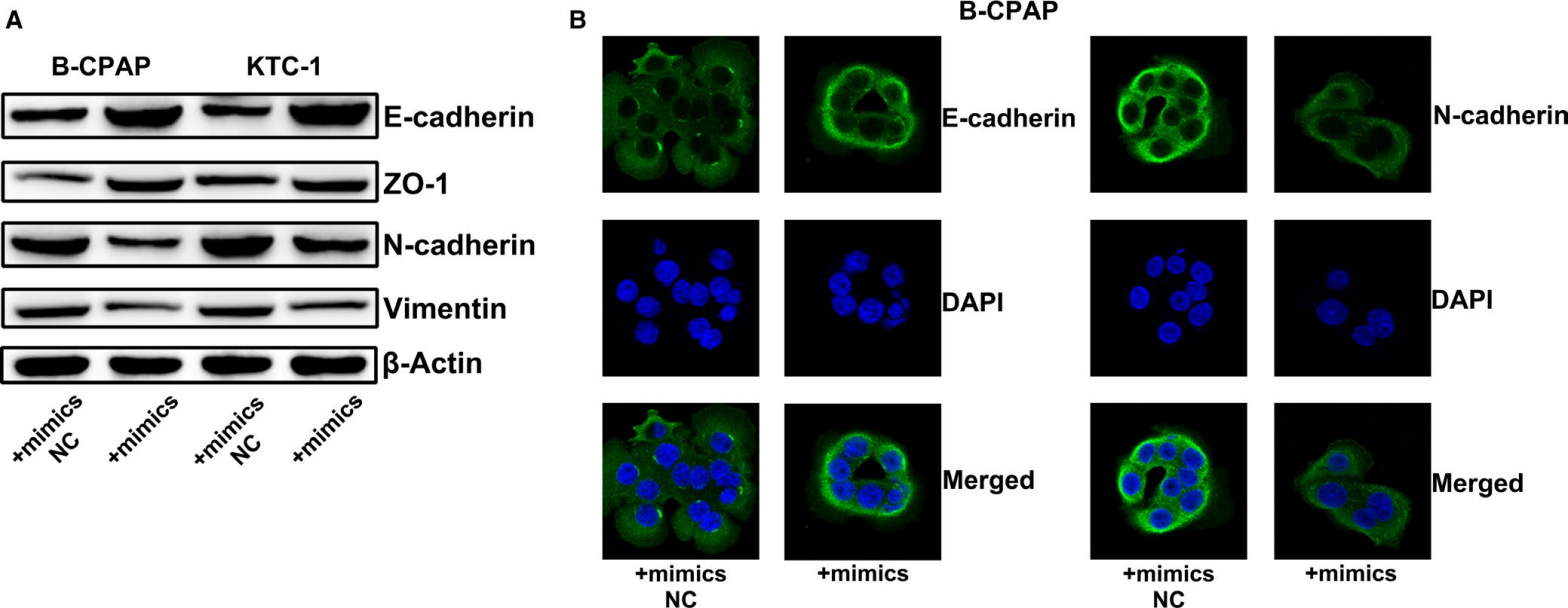
miR‐451a inhibits epithelial‐mesenchymal transition of papillary thyroid cancer cells. A, Western blotting assay of epithelial‐mesenchymal transition markers in B‐CPAP or KTC‐1 cells transfected with miR‐451a mimics or mimics NC. B, Immunofluorescence assay of epithelial‐mesenchymal transition markers in B‐CPAP cells transfected with miR‐451a mimics or mimics NC; NC for negative control

Based on the reported differential expression of miR‐451a in PTC patients with or without lymph node metastasis, we investigate the correlation between miR‐451a and epithelial‐mesenchymal transition. Western blotting and immunofluorescence assay of the epithelial‐mesenchymal transition markers showed that overexpression of miR‐451a suppressed the epithelial‐mesenchymal transition of PTC cancer cells (Figures 2A2, 3, 4B).

**Figure 3:**
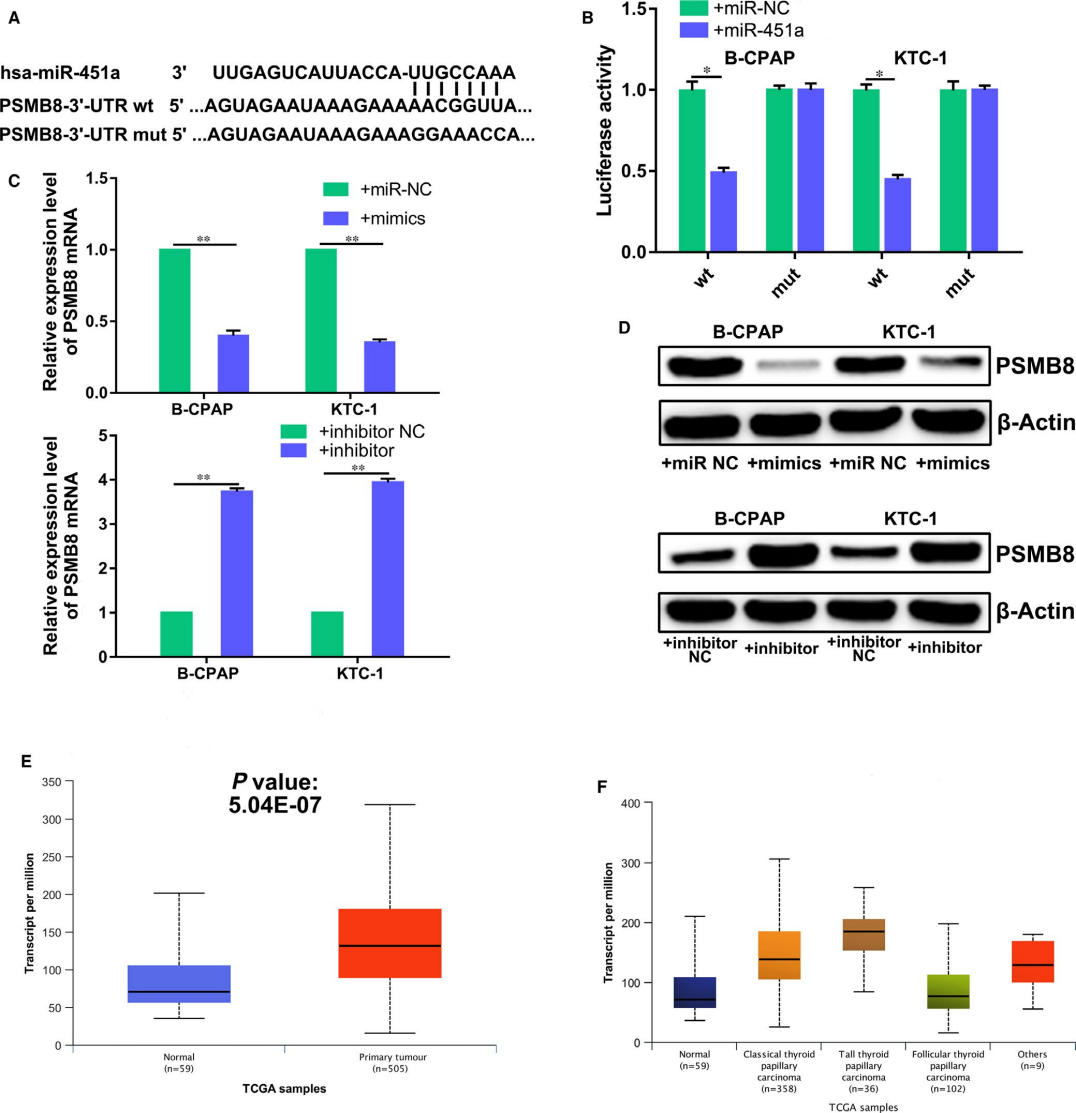
Proteasome subunit beta type‐8 is a direct target gene of miR‐451a in papillary thyroid cancer cells. A, Schematics of miR‐451a, PSMB8 wild‐type (WT) and mutant (Mut) luciferase reporter plasmids. B, Analysis of luciferase activation in treated B‐CPAP or KTC‐1 cells. C, The effects of miR‐451a on the mRNA expression of PSMB8, by RT‐qPCR. D, The effects of miR‐451a on the protein expression of PSMB8, by Western blotting. E, The expression level of PSMB8 in thyroid cancer tissues and paracancerous tissues, based on the data of The Cancer Genome Atlas. F, The expression level of PSMB8 in different histological type of thyroid cancer tissues, based on the data of The Cancer Genome Atlas; NC for negative control, *P* < .05 marked *, *P* < .01 marked ** and *P* < .001 marked ***

**Figure 4:**
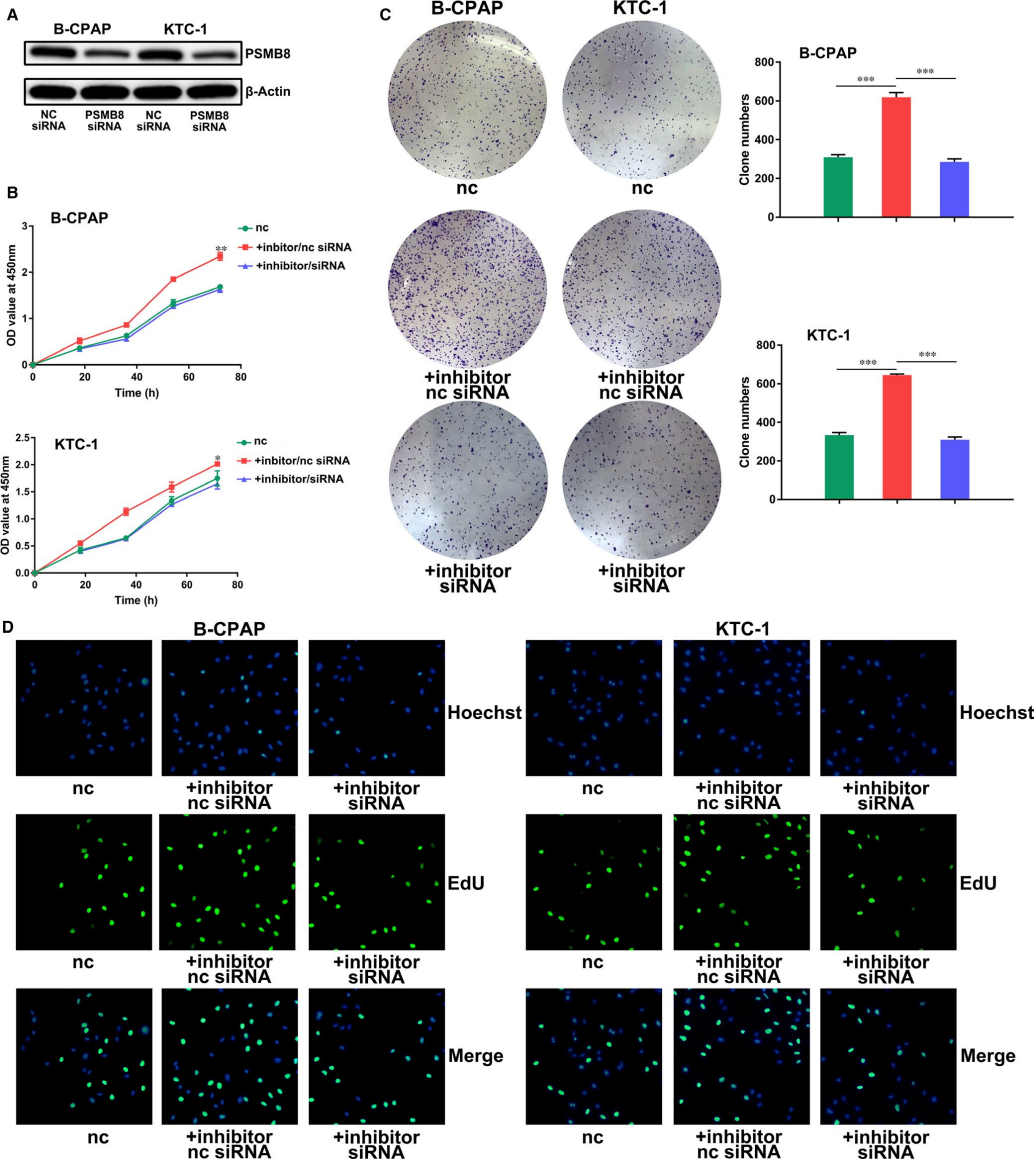
miR‐451a inhibits proliferation of papillary thyroid cancer cells by suppressing PSMB8. A, Knockdown of PSMB8 expression in B‐CPAP or KTC‐1 cells by transfection of PSMB8 siRNA. B, CCK‐8 assay of B‐CPAP or KTC‐1 cells transfected with miR‐451a inhibitor/PSMB8 siRNA or inhibitor/nc siRNA. C, Clone formation assay of B‐CPAP or KTC‐1 cells transfected with miR‐451a inhibitor/PSMB8 siRNA or inhibitor/nc siRNA. D, EdU DNA cell proliferation assay of B‐CPAP or KTC‐1 cells transfected with miR‐451a inhibitor/PSMB8 siRNA or inhibitor/nc siRNA; nc for negative control, *P* < .05 marked *, *P* < .01 marked ** and *P* < .001 marked ***

### 3.3 Proteasome subunit beta type‐8 is a direct target gene of miR‐451a in papillary thyroid cancer cells

We predicted the potential target genes of miR‐451a by bioinformatics analysis in order to further illustrate the molecular mechanisms underlying the tumour suppressor activity of miR‐451a. We identified and chose PSMB8 for further analysis (Figure 3A). Luciferase reporter assay indicated that PSMB8 is a target gene of miR‐451a, as predicted (Figure 3B). The results showed that the luciferase activity of the plasmid carrying wild‐type PSMB8 3′‐UTR, but not the mutant type, was significantly decreased by miR‐451a overexpression in B‐CPAP and KTC‐1 cells. Furthermore, RT‐qPCR and Western blotting showed that miR‐451a overexpression reduced PSMB8 expression while miR‐451a knockdown increased PSMB8 expression in B‐CPAP and KTC‐1 cells, at both the mRNA and protein levels (Figure 3C,D). These results suggest that PSMB8 is a direct target gene of miR‐451a in papillary thyroid cancer cells. We further investigated the expression profile of PSMB8 in thyroid cancer. Expression profile data from The Cancer Genome Atlas showed that the expression level of PSMB8 is higher in thyroid cancer tissues than paracancerous tissues (Figure 3E,F). These data suggested that miR‐451a may act as a tumour suppressor by targeting PSMB8.

### 3.4 miR‐451a inhibits proliferation, epithelial‐mesenchymal transition and induces apoptosis of papillary thyroid cancer cells by suppressing PSMB8 expression

**Figure 5:**
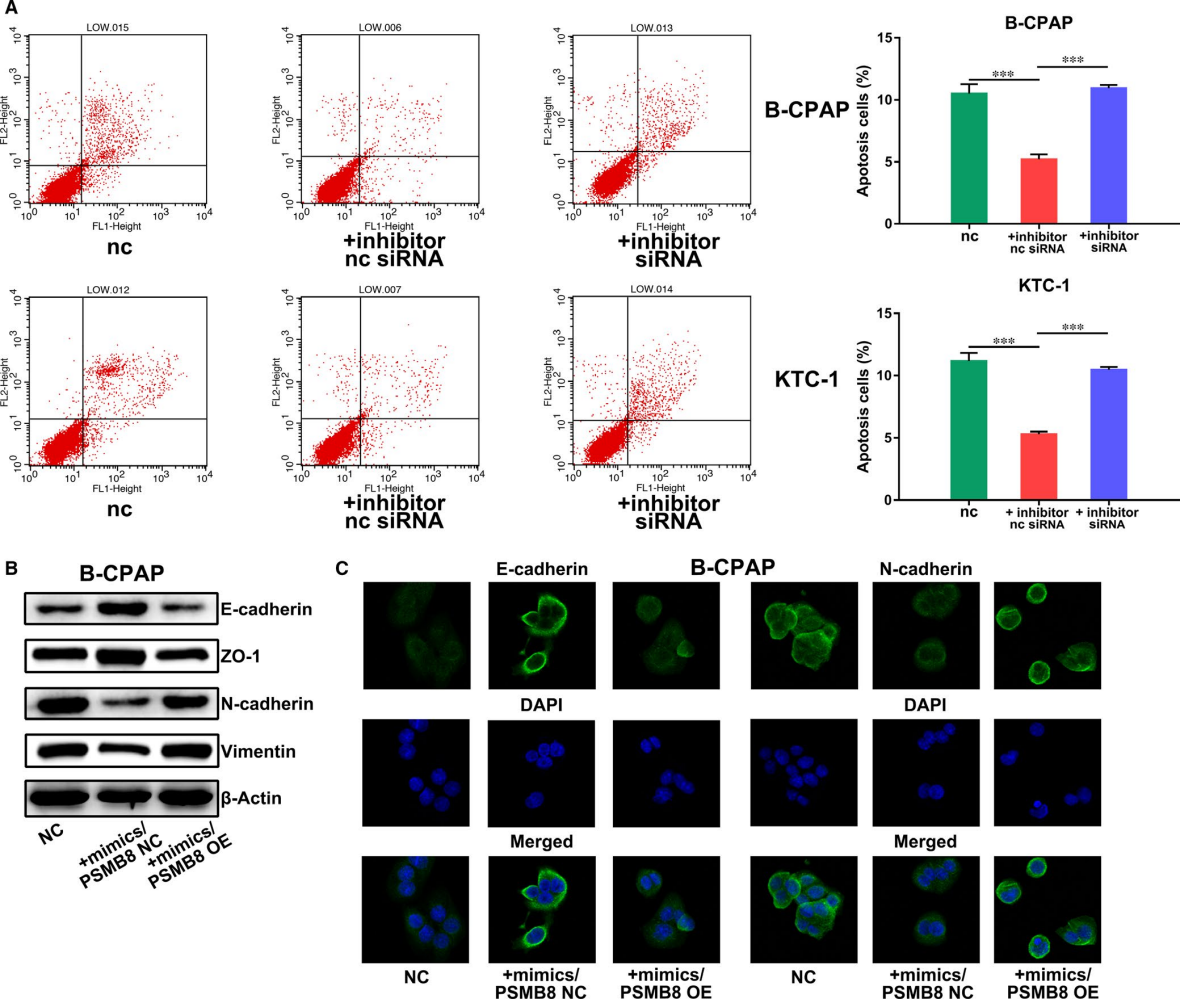
miR‐451a inhibits epithelial‐mesenchymal transition and induces apoptosis of papillary thyroid cancer cells by suppressing PSMB8. A, Apoptosis assay of B‐CPAP or KTC‐1 cells transfected with miR‐451a inhibitor/PSMB8 siRNA or inhibitor/nc siRNA. B, Western blotting assay of epithelial‐mesenchymal transition markers in B‐CPAP cells transfected with miR‐451a mimics/PSMB8 OE or mimics/PSMB8 NC. C, Immunofluorescence assay of epithelial‐mesenchymal transition markers in B‐CPAP cells transfected with miR‐451a mimics/PSMB8 OE or mimics/PSMB8 NC; NC for negative control, OE for overexpression, *P* < .05 marked *, *P* < .01 marked ** and *P* < .001 marked ***

We further investigated whether the aforementioned functions of miR‐451a were achieved by suppressing PSMB8. We decreased the expression of miR‐451a by inhibitor transfection, while decreasing the expression of PSMB8 by siRNA transfection (Figure 4A). We compared the experimental group with a normal control group and an inhibitor/nc siRNA group. In vitro functional experiments showed that the inhibitor/siRNA group showed similar proliferation capabilities with the control group, while the inhibitor/nc siRNA group showed increased proliferation capabilities and suppressed apoptosis (Figures 4B‐D and 5A). We also increased the expression level of miR‐451a by mimics transfection while increasing the expression of PSMB8 by overexpression plasmid transfection (Figure S1C). We compared the expression of epithelial‐mesenchymal transition markers between experimental group with a normal control group and an mimics/overexpression plasmid NC group. Results of Western blotting and immunofluorescence assay showed that cells treated with mimics/overexpression plasmid NC showed suppressed epithelial‐mesenchymal transition while cells treated with mimics/overexpression plasmid reversed the aforementioned suppression of epithelial‐mesenchymal transition (Figure 5B,C).

## 4 DISCUSSION

Several studies have reported the cancer suppressor activity of miR‐451a in other cancer types.^21^, ^22^, ^23^ miR‐451a was reported to inhibit aggressiveness features of lung squamous cell cancer by targeting KIF2A.^24^ In colorectal cancer, miR‐451a was reported to inhibit proliferation and increase apoptosis by targeting BAP31.^25^ Plasma exosomal miR‐451a was reported to be a novel biomarker for early prediction and recurrence of non‐small‐cell lung cancer.^26^ In the field of PTC research, though several studies have reported the down‐regulation of miR‐451a in PTC/healthy participants and PTC patients with lymph node metastasis/PTC patients without lymph node metastasis, these studies did not study the underlying molecular mechanism. One of the published papers reported that miR‐451a could inhibit the proliferation of PTC cells in vitro. However, this conclusion was based on merely one in vitro CCK‐8 assay which requires further validation with other in vitro and in vivo experiments. Therefore, in this study, we studied the underlying mechanism of the cancer suppressor functions of miR‐451a in PTC. In vitro and in vivo functional experiments showed that miR‐451a could suppress the oncogenetic capabilities of PTC cells by targeting PSMB8. PSMB8 was reported to be a target gene of miR‐451a in A549 lung cancer cells.^27^ Strong expression of PSMB8 was also reported to be correlated with increased depth of local invasion and lymph node metastasis in gastric cancer.^28^ Based on these reported results and our own results, we suggested that miR‐451a may act as a cancer suppressor by inhibiting the cancer promoter function of PSMB8 in PTC. This miR‐451a/PSMB8 axis may serve as novel therapy targets for PTC patients.

## CONFLICT OF INTEREST

The authors confirm that there are no conflicts of interest.

## AUTHOR CONTRIBUTIONS

YZ designed the study and review the paper, XF performed the experiments, analysed the data and wrote the paper.

## Data Availability

The data that support the findings of this study are available from the corresponding author upon reasonable request.
