# Supplementary figures and images for: miR‐451a inhibits cancer growth, epithelial‐mesenchymal transition and induces apoptosis in papillary thyroid cancer by targeting PSMB8
Source: J Cell Mol Med. 2019 Sep 27;23(12):8067–75. doi: 10.1111/jcmm.14673 (PMC6850967; doi:10.1111/jcmm.14673)

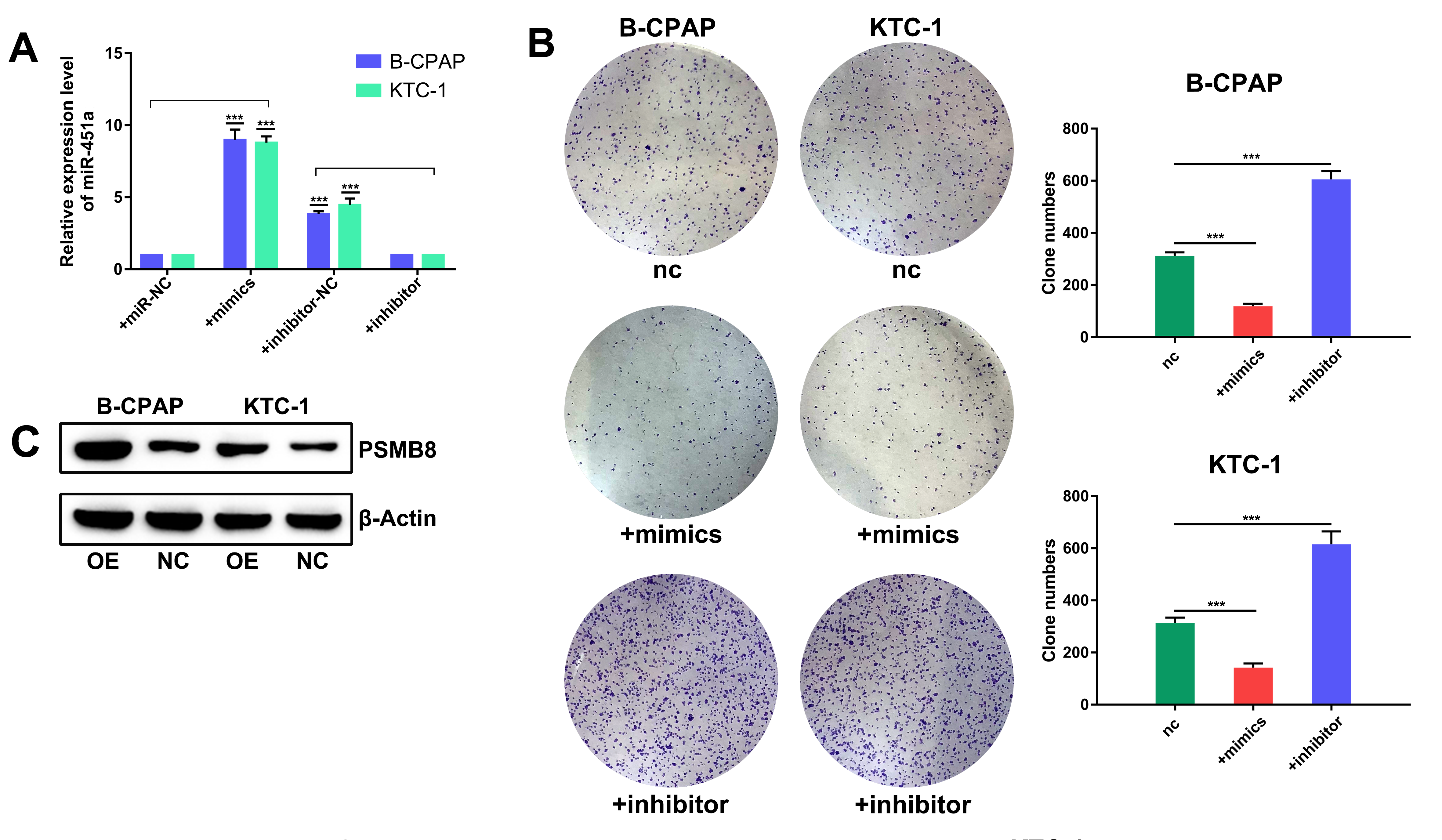

Supplement: Supplementary file 1 [file JCMM-23-8067-s001.tif]
